# Contact Efficacy of Two Amorphous Silica Powders against the Red Flour Beetle, *Tribolium castaneum* (Herbst) (Coleoptera: Tenebrionidae)

**DOI:** 10.3390/insects14110833

**Published:** 2023-10-24

**Authors:** Selladurai Manivannan, Bhadriraju Subramanyam

**Affiliations:** Department of Grain Science and Industry, Kansas State University, Manhattan, KS 66506, USA; sbhadrir@ksu.edu

**Keywords:** amorphous silica powders, *T. castaneum*, adult mortality, progeny production, particle size

## Abstract

**Simple Summary:**

In recent years, there has been a resurgence of interest in silica-based powders as a non-chemical alternative for safeguarding stored food grains from stored product insects. Previous research has indicated that the red flour beetle, *Tribolium castaneum* (Herbst) (Coleoptera: Tenebrionidae), displays a higher tolerance to inert dusts. Nevertheless, the effectiveness of the inert dusts may be influenced by their elemental composition and physical characteristics. To investigate this further, we conducted contact tests in concrete arenas to evaluate the efficacy of two amorphous silica powders, labeled as silica powder 1 and silica powder 2, which were obtained from Imery’s Chemicals in Lompoc, CA, USA. Our findings revealed that exposing *T. castaneum* adults to silica powder 1, characterized by smaller particle sizes, was significantly more successful in inducing adult mortality and inhibiting the production of adult progeny at lower test concentrations compared to silica powder 2. Our results highlight the potential of these silica powders as a valuable tool for the control of *T. castaneum* for treating empty bin floors prior to the storage of newly harvested grains.

**Abstract:**

The contact efficacy of two amorphous silica powders 1 and 2 procured from Imery’s chemicals, Lompoc, CA, USA, were evaluated against the red flour beetle, *Tribolium castaneum* (Herbst). The efficacy of the silica two powders was evaluated by exposing 10 adults of *T. castaneum* to twelve different concentrations of silica powder 1 and 2 for 12, 24, 36, and 48 h. Mortality assessments were made after 14 d, and data on adult progeny production were recorded at 42 d. Complete mortality of *T. castaneum* was observed when adults were exposed for 36 h to concentrations of 1.5 to 5 g/m^2^ of silica powder 1. Conversely, in tests with silica powder 2, complete mortality was only achieved when adults were exposed for 48 h to concentrations ranging from 0.75 to 5 g/m^2^. Silica powder 1 exhibited greater efficacy in inhibiting adult progeny production in *T. castaneum*, particularly at a concentration of 2.0 g/m^2^ after 24 h exposure. Overall, silica powder 1 displayed superior performance in terms of adult mortality and the suppression of *T. castaneum* adult progeny production. This advantage can be attributed to the smaller particle size of silica powder 1 when compared to silica powder 2.

## 1. Introduction

The development of insect resistance to traditional insecticides and the increasing demand for insecticide food has increased the search for viable alternatives that are safer for application in stored product environments. Among various non-chemical alternatives used for safeguarding stored food products, there has been a renewed focus on the use of inert dusts in recent times. Inert dusts are dry powders that are chemically unreactive [1], and are now an integral part of the integrated pest management of stored product insects [2]. Inert dusts exhibit strong insecticidal activity with low mammalian toxicity [3,4,5]. Inert dusts applied in stored product environments have been shown to augment the efficacy of other pest management methods such as aeration, fumigation, and heat treatments, with success stories reported in Australia and USA [6,7]. Inert dusts, specifically diatomaceous earth and zeolites, are considered as viable alternatives to chemical insecticides [1,8]. Many studies have documented the effectiveness of inert dusts on stored product insects when admixed with grains [9,10,11,12,13,14,15,16,17]. Newer formulations of diatomaceous earths are very effective on stored product insects compared to older formulations which required higher application rates, often affecting the physical properties of grains [18,19]. However, studies pertaining to the use of inert dusts for surface treatments, including bins walls and floors, are very limited [8].

Silica dusts containing amorphous silica exhibit remarkable efficiency at lower application rates when compared to other formulations [18] and hence are used for structural treatments. Inert dusts affect insects by absorbing the lipids from the insects’ epicuticle, ultimately resulting in death by desiccation [1,18,20,21,22,23,24,25]. The susceptibility of stored product insects to diatomaceous earth is known to vary among species [18,19,26,27]. Stored product insects such as the *Cryptolestes* species are generally more susceptible to diatomaceous earth products, while the *Sitophilus* species are slightly less susceptible, whereas the *Oryzaephilus* species, the lesser grain borer, *Rhyzopertha dominica* (F.), and flour beetles, the *Tribolium* species, are the more tolerant ones [18,27,28,29]. Similarly, various researchers have indicated flour beetles, *Tribolium* spp., as the most tolerant insect species among stored product insects to inert dusts [27,30,31,32]. Yao [33] evaluated the efficacy of a synthetic zeolite against five stored product insects such as *R. dominica*, rice weevil, *Sitophilus oryzae* (L.), maize weevil, *Sitophilus zeamais* (Motschuslky), sawtoothed grain beetle, *Oryzaephilus surinamensis* (L.), and red flour beetle, *Tribolium castaneum* (Herbst). In his research, it was discovered that among the five species exposed to fine zeolite-treated concrete arenas at both 5 g/m^2^ and 10 g/m^2^, *T. castaneum* adults were the least susceptible. He observed that a minimum of 24 h exposure was needed to observe a 100% mortality of *T. castaneum* adults at the two tested concentrations.

The diatomaceous earth formulation, Protect-It, registered for use on raw grains and on floor surfaces, has shown to manage many stored product insects [3,18]. The surface application of Protect-It at a labelled rate of 0.5 g/m^2^ required an exposure time of 48 or 72 h to achieve complete mortality of *T. castaneum*, and the confused flour beetle, *Tribolium confusum* Jacquelin du Val, when the adults were not provided food after treatment [34]. A recent study from Greece [8] showed that *T. confusum* adults exposed to two diatomaceous earths, DE5 and DE 6, and zeolite-treated concrete surfaces at 0.5 and 1.0 g/m^2^, achieved complete mortality of adults after 3 days of exposure. In contact efficacy bioassays conducted in concrete arenas [35], DiaFil^®^ 610 demonstrated 100% control of *T. castaneum* at application rates of 2.5 and 5.0 g/m^2^ after a 24 h exposure period. These studies indicate *Tribolium* species as one of the tolerant insect groups to inert dusts, and any research on inert dust formulations showing better efficacy on *T. castaneum* at lower application rates can be crucial in the effective management of *T. castaneum*. Moreover, the insecticidal activity of inert dusts can vary based on the geographical location, diatom species, pH, particle size distribution, internal surface area, and lipid adsorption capability [36,37]. Studies by Vayias et al. [38] showed that the smaller diatomaceous earth particles exhibit better insecticidal activity on beetle species. In this study, two candidate amorphous diatomaceous earths, supplied by Imery’s chemicals, Lompoc, CA, USA, referred to here as silica powders 1 and 2, were evaluated for their contact efficacy against *T. castaneum* using concrete arenas to simulate empty bin floors. The objective of this study was to determine the effect of silica powders 1 and 2 on adult mortality and progeny production of *T. castaneum* exposed for different concentrations and exposure periods.

## 2. Materials and Methods

### 2.1. Silica Powders

Two amorphous silica powders, 1 and 2, with different elemental composition, size, and shape characteristics, supplied by Imery’s chemicals, Lompoc, CA, USA, were used for the contact bioassays against *T. castaneum*. The elemental composition, particle size diameter, and shape characteristics of the two silica powders have been described in detail elsewhere (Manivannan and Subramanyam; accepted for publication in *Journal of Stored Products Research*). The silicon dioxide content in silica powder 1 was 94% and 97% for silica powder 2. The particles with diameters of 26.4 and 31.4 µm dominated the silica powder 1 and silica powder 2, respectively (Manivannan and Subramanyam, unpublished data).

### 2.2. Test Insects

Insects used in the experiments originated from colonies maintained in the Department of Grain Science and Industry, Kansas State University, Manhattan, KS, USA, since 1999. Insect cultures of *T. castaneum* (laboratory strain) were reared in 0.94 L glass mason jars containing 250 g of organic whole wheat flour (Great River Organic Milling Company, Fountain City, WI, USA) supplemented with 5% brewer’s yeast (by weight). The mason jars were secured with metal screens (250 µm) and filter paper lids (9 cm diameter) and placed inside a growth chamber under 28 °C and 65% r.h. with a 14:10 light:dark photoperiod.

### 2.3. Concrete Arenas

Rockite concrete mix (Rockite, Hartline Products Co., Inc., Cleveland, OH, USA) was used to make into a slurry using tap water mixed at 2:1 ratio. Concrete dishes were prepared by pouring the slurry into plastic Petri dishes of 9 cm diameter and 1.5 cm height (Fisher Scientific Co., LLC., Pittsburgh, PA, USA). The concrete-poured dishes were placed on a laboratory workbench allowing them to dry for 24 h. Following drying, the inner walls of the dishes were coated with a layer of polytetrafluoroethylene (Insect-a-Slip, BioQuip product, Inc, Rnach, Domnigeuz, CA, USA) to prevent insects from crawling on the sides of the dishes, thus avoiding contact with treated arenas [39,40]. The concrete arenas were used to simulate empty bin or silo floors.

### 2.4. Contact Efficacy

Three different tests were carried out to determine the effect of silica powders 1 and 2 on *T*. *castaneum* adults. In the first test, the concrete arenas were treated with silica powders 1 and 2 at twelve different concentrations: 0 (untreated control), 0.5, 0.75, 1.0, 1.5, 2.0, 2.5, 3.0, 3.5, 4.0, 4.5, and 5.0 g/m^2^. After adding the required concentration of powders onto the dishes, a dissection needle was used to distribute the powders on the concrete arenas. Ten unsexed 1-4-week-old adults of *T. castaneum* were introduced to concrete arenas at each concentration of the silica powders for 12, 24, 36, and 48 h. There were five replications for each powder concentration–exposure combination. The concrete arenas were secured with lids and then placed inside the growth chamber at rearing conditions. After the intended exposure period, insects from each concrete arena were carefully transferred to individual 150 mL round-bottom plastic containers containing 30 g of whole wheat flour and 5% brewer’s yeast. After 14 d, the wheat flour was sieved using 595 µm sieve (US standard sieve 30) openings to separate the adults from flour. The data on insect mortality were determined by recording the number of dead insects from the total exposed. Insects that remained immobile when poked with a camel’s hairbrush were considered dead.

In the second test, the effect of silica powders 1 and 2 on the progeny production of *T. castaneum* was evaluated after 42 d. The protocol followed was similar to those mentioned in the first test. At 42 d, the adults were removed by sieving the diet using 595 µm sieve openings. The number of progeny production data was recorded by subtracting the ten parental adults from the total progeny counts.

In the third test, ten unsexed 1–4-week-old adults of *T. castaneum* were exposed to 5.0 g/m^2^ and 6.0 g/m^2^ of silica powder 1 and silica powder 2, respectively, for 1, 2, 4, 6, 8, 10, 12, 14, 16, 18, 20, 22, and 24 h. Five replications were maintained for each powder–concentration exposure period combination. In the third test, adults after intended exposure were handled similar to first and second tests including mortality assessment and adult progeny production data. Separate arenas were used for mortality and progeny production.

### 2.5. Data Analysis

Control mortality was absent across all exposure times and, therefore, the mortality data were not corrected for control mortality. The non-linear model, *y* = *a* + *be*^−*x*^, was fitted to the percentage mortality data of *T. castaneum* as a function of concentration of silica powder 1 and silica powder 2 for different exposure periods where possible, using Table Curve2D software, Version 5.01.01 (Jandel Scientific, San Rafael, CA, USA). The non-linear model, ln*y* = *a* + *bx*^0.5^, was fitted to the progeny production data relative to the concentration of silica powders 1 and 2 at varying exposure durations where feasible using Table Curve2D software, Version 5.01.01. The non-linear model, *y*^0.5^ = *a* + *bx*^0.5^, was fitted to the adult mortality and progeny production data relative to the exposure period for silica powder 1 at 5.0 g/m² and silica powder 2 at 6.0 g/m². The non-linear model, *y*^0.5^ = *a + bx*^0.5^, was fitted to adult mortality and progeny production data as a function of exposure times for silica powder 1 at 5.0 g/m^2^ and silica powder 2 at 6.0 g/m^2^. The values for parameters *a* and *b* were determined by fitting equations to the data on adult mortality and progeny production relative to both concentration and exposure period. Each conceivable pairwise comparison between the silica powders and their respective exposure periods was carried out by comparing individual models with pooled models [41]. Any two models were considered statistically significant from one another (*p* < 0.05) when the *F*-test revealed that individual models differed from the pooled model. The data analysis was performed using SAS 9.4 software [42]. The graphs were generated using SigmaPlot 12.5 software (Systat, Software, Inc., San Jose, CA, USA).

## 3. Results

### 3.1. Responses of T. castaneum Adults to Silica Powders

The mean ± SE mortality of *T. castaneum* adults treated with 0.5 to 5 g/m^2^ of silica powder 1 in concrete arenas for 12, 24, 36, and 48 h ranged from 12 ± 5 to 100%. The minimum concentrations required for complete mortality of *T. castaneum* exposed to silica powder 1 for 24, 36, and 48 h were 5.0, 1.5, and 1.5 g/m^2^, respectively. On the other hand, complete mortality of *T. castaneum* adults was not achieved at any of the tested concentrations when they were exposed for 12 h to 0.5 to 5 g/m^2^ of silica powder 1 (Table 1). The mean ± SE mortality of *T. castaneum* adults treated with 0.5 to 5 g/m^2^ of silica powder 2 for 12, 24, 36, and 48 h ranged from 14 ± 5 to 100%. None of the tested concentrations resulted in complete mortality of *T. castaneum* when adults were treated with 0.5 to 5 g/m^2^ of silica powder 2 for 12, 24, and 36 h. However, the minimum concentration required for 100% mortality of adults exposed for 48 h to concrete arenas treated with silica powder 2 was 0.75 g/m^2^ (Table 1).

**Figure 1:**
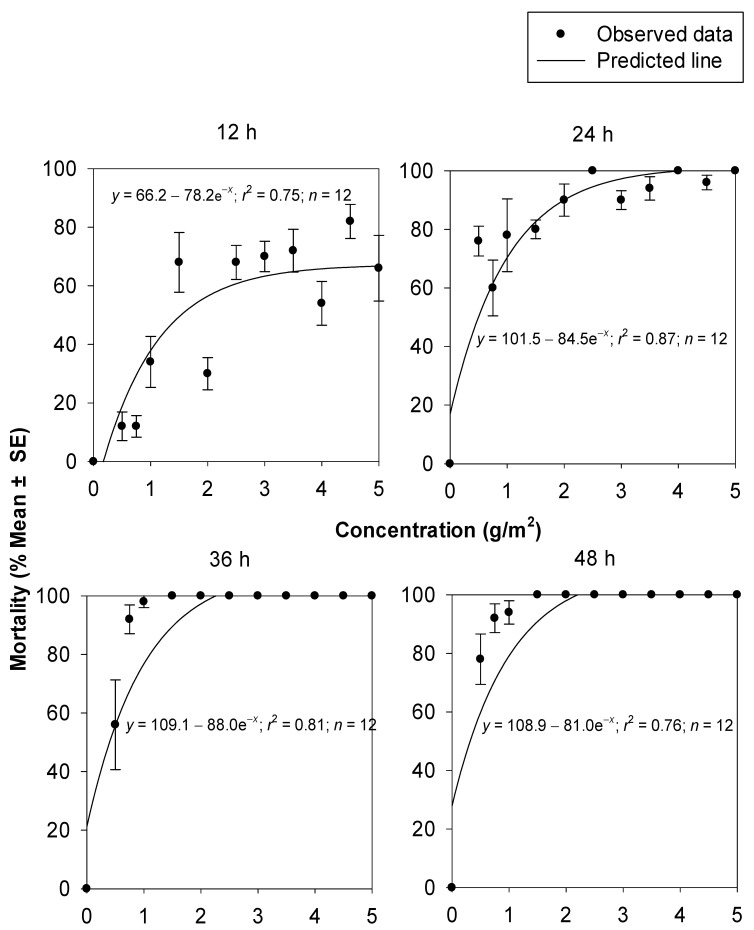
Percentage mortality of *T. castaneum* adults at 14 d after exposure to concrete arenas treated with silica powder 1 at different concentrations and exposure times.

**Figure 2:**
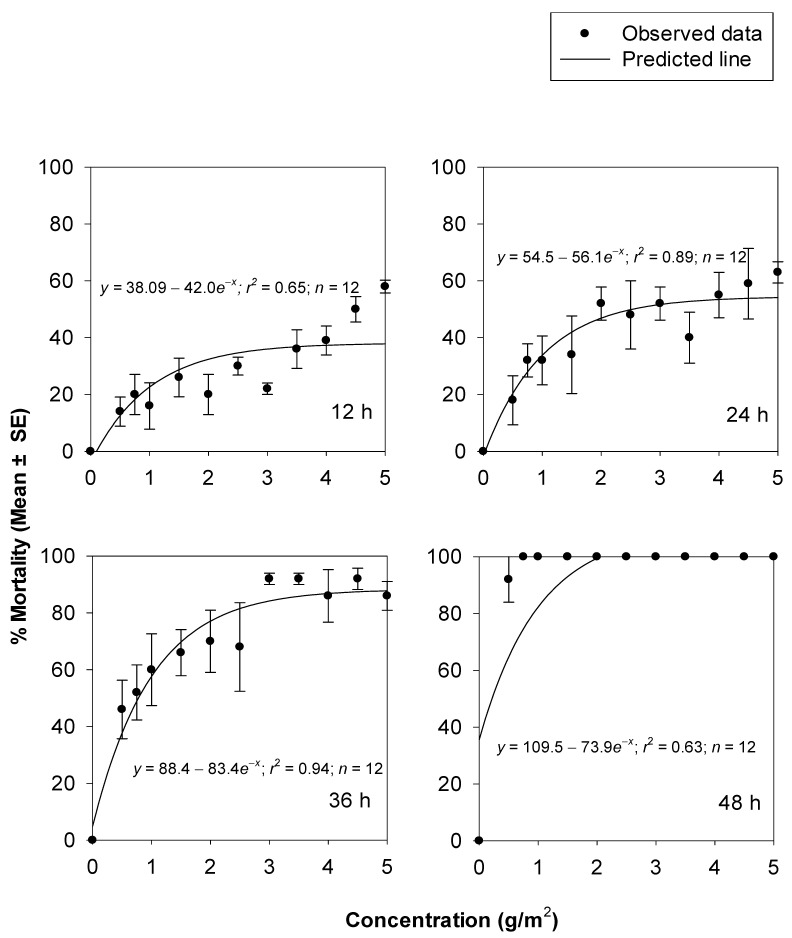
Percentage mortality of *T. castaneum* adults at 14 d after exposure to concrete arenas treated with silica powder 2 at different concentrations and exposure times.

The increase in the percentage mortality of *T. castaneum* at increasing concentrations of silica powder 1 and 2 were non-linear. The fitted models effectively described the relationship between the mortality data of *T. castaneum* and the concentrations of silica powder 1 (*r*^2^, _range_ = 0.75–0.87) (Figure 1) and silica powder 2 (*r*^2^, _range_ = 0.63–0.94) (Figure 2), at 12, 24, 36, and 48 h. Comparisons of the non-linear models fitted for percentage insect mortality were significantly higher for silica powder 1 compared to silica powder 2 after 12 (*F* = 9.92; df = 2, 20; *p* = 0.0010), 24 (*F* = 64.79; df = 2, 20; *p* = 0.0000), and 36 h (*F* = 12.24; df = 2, 20; *p* = 0.0003). This indicates that silica powder 1 was more efficacious against *T. castaneum* adults compared to silica powder 2.

The non-linear model fitted to the percentage insect mortality data of silica powder 1 was not significantly different compared to silica powder 2 after 48 h exposure (*F* = 0.10; df = 2, 20; *p* = 0.9009). The mortality responses of *T. castaneum* exposed to silica powder 1 at 24 and 36 h (*F* = 0.95; df = 2, 20; *p* = 0.4047), 24 and 48 h (*F* = 1.29; df = 2, 20; *p* = 0.2976), and 36 and 48 h (*F* = 0.10; df = 2, 20; *p* = 0.9072) were not significantly different. However, all the other pairwise comparisons between exposure times for silica powder 1 (*F*, _range_ = 16.41–24.17; df = 2, 20; *p* = 0.0000) and silica powder 2 (*F*, _range_ = 7.81–56.70; df = 2, 20; *p*, _range_ = 0.0000–0.0031) were significant (Table 2).

**Figure 3:**
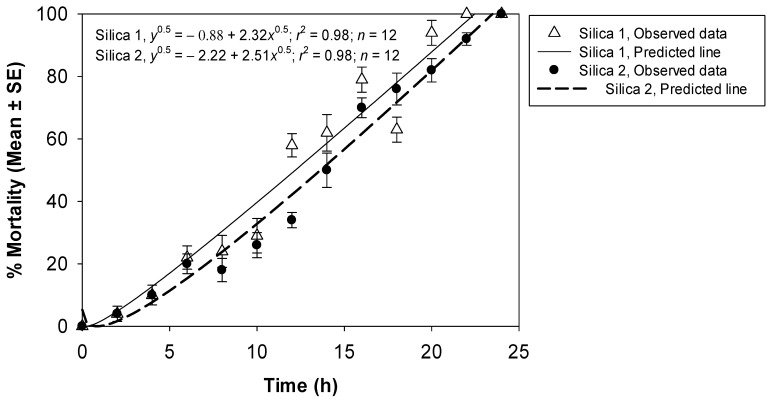
Percentage mortality of *T. castaneum* adults at 14 d after exposure to concrete arenas treated with 5.0 g/m^2^ of silica powder 1 and 6.0 g/m^2^ of silica powder 2 for various exposure times.

Complete mortality was observed when *T. castaneum* adults were exposed to 5.0 g/m^2^ of silica powder 1 for 22 h. Complete mortality of the adults was observed only after exposure for 24 h to 6.0 g/m^2^ of silica powder 2. The fitted models effectively described the association between the percentage mortality data and the exposure period of silica powder 1 (*r*^2^ = 0.98) and silica powder 2 (*r*^2^ = 0.98) (Figure 3). A comparison of the non-linear models fitted for the percentage insect mortality data as a function of the exposure period showed a significant difference between silica powder 1 and 2 (*F* = 10.41; df = 2, 22; *p* = 0.0007).

### 3.2. Adult Progeny Production

The mean ± SE adult progeny production observed in the controls ranged from 77.2 ± 6.6 to 96.4 ± 9.8. The adult progeny produced after exposure to 0.5 to 5.0 g/m^2^ of silica powder 1 for 12 to 48 h ranged from 0 to 39.8 ± 7.3. The minimal concentrations needed to completely suppress adult progeny production of *T. castaneum* when adults were exposed to concrete arenas treated with silica powder 1 for 24, 36, and 48 h were 2, 0.75, and 0.75 g/m², respectively. However, the complete inhibition of adult progeny production of *T. castaneum* was not attained in any of the tested concentrations when they were exposed to 0.5 to 5 g/m^2^ of silica powder 1 for 12 h (Table 3). The adult progeny produced after exposure to 0.5 to 5.0 g/m^2^ of silica powder 2 ranged from 0 to 67.2 ± 9.3. The minimum concentrations required to achieve complete inhibition of adult progeny production of *T. castaneum* after exposure to silica powder 2 for 36 and 48 h was 3 and 0.75 g/m^2^, respectively. However, complete inhibition of adult progeny production of *T. castaneum* was not attained at any of the tested concentrations when they were exposed to 0.5 to 5 g/m^2^ of silica powder 1 for 12 and 24 h (Table 3).

**Figure 4:**
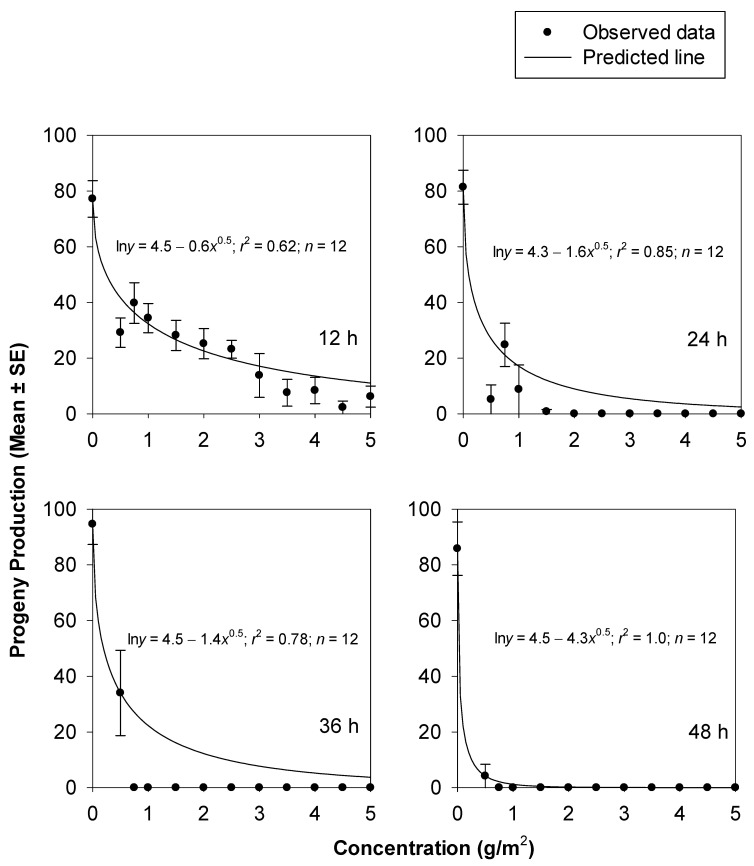
Adult progeny production from *T. castaneum* adults at 42 d after exposure to concrete arenas treated with silica powder 1 at different concentrations and exposure times.

**Figure 5:**
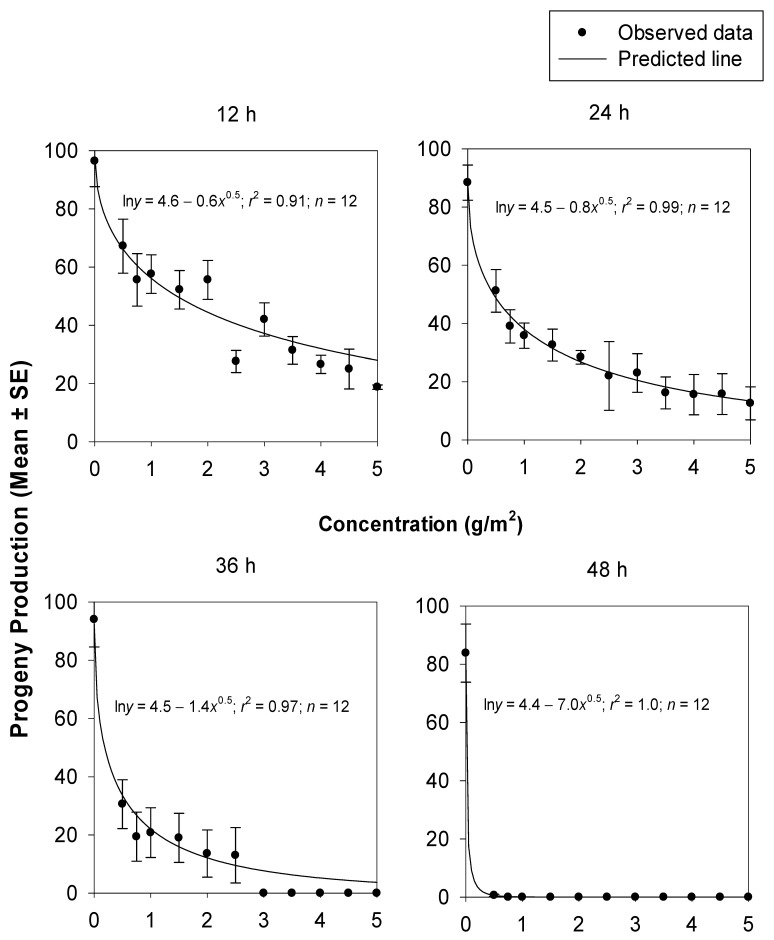
Adult progeny production from *T. castaneum* adults at 42 d after exposure to concrete arenas treated with silica powder 2 at different concentrations and exposure times.

The decreases in the adult progeny production of *T. castaneum* at increasing concentrations of silica powder 1 and 2 were non-linear. The fitted models successfully described the relationship between the adult progeny production of *T. castaneum* and the concentrations of silica powder 1 (*r*^2^, _range_ = 0.62–1.0) (Figure 4) and silica powder 2 (*r*^2^, _range_ = 0.91–1.0) (Figure 5) after 12, 24, 36, and 48 h. The comparisons of the non-linear models fitted for adult progeny production were significantly higher for silica powder 2 compared to silica powder 1 after 12 (*F* = 44.17; df = 2, 20; *p* = 0.0000), 24 (*F* = 55.82; df = 2, 20; *p* = 0.0000), 36 h (*F* = 5.00; df = 2, 20; *p* = 0.0174), and 48 h (*F* = 30.04; df = 2, 20; *p* = 0.0000). This indicated that silica powder 1 was more effective against *T. castaneum* adults compared to silica powder 2 in inhibiting adult progeny production. The mortality responses of *T. castaneum* exposed to silica powder 1 at 24 and 36 h were not significantly different (*F* = 0.07; df = 2, 20; *p* = 0.9294). However, all the other pairwise comparisons for silica powder 1 (*F*, _range_ = 12.75–151.54; df = 2, 20; *p* = 0.0000–0.0002) and silica powder 2 (*F*, _range_ = 30.15–2173.74; df = 2, 20; *p* = 0.0000) were significant (Table 4).

**Figure 6:**
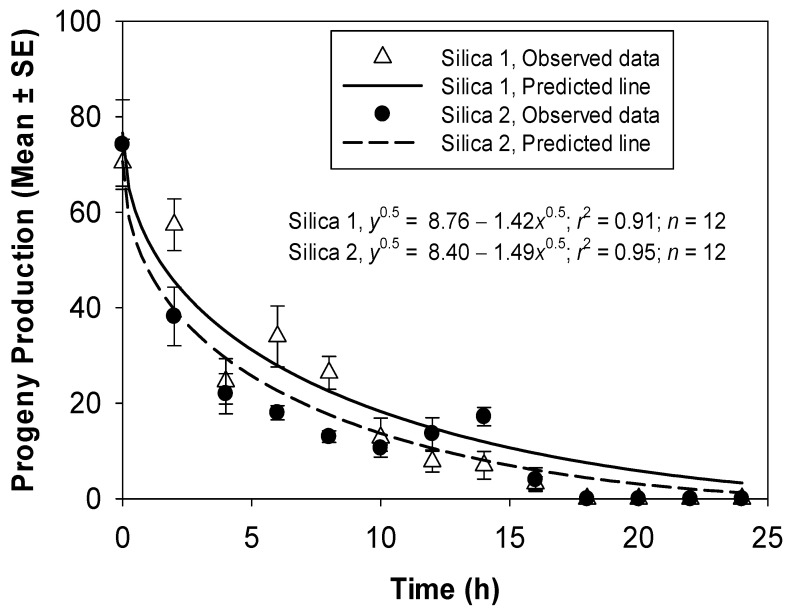
Adult progeny production from *T. castaneum* adults at 42 d after exposure to concrete arenas treated with 5.0 g/m^2^ of silica powder 1 and 6.0 g/m^2^ of silica powder 2 for various time periods.

Complete inhibition in adult progeny production of *T. castaneum* was observed in the third test when adults were treated with 5.0 g/m^2^ of silica powder 1 for 18 to 24 h. Complete inhibition in progeny production was also observed when adults were exposed to 6.0 g/m^2^ of silica powder 2 for 18 to 24 h. The fitted models successfully elucidated the relationship between adult progeny production and the exposure period of silica powder 1 (*r*^2^ = 0.91) and silica powder 2 (*r*^2^ = 0.95) (Figure 6). Comparisons of the non-linear models fitted for the adult progeny production data as a function of the exposure period showed no significant difference between silica powder 1 and 2 (*F* = 0.94; df = 2, 22; *p* = 0.4063).

## 4. Discussion

Many studies have investigated the effectiveness of inert dusts against the adults of *T. castaneum* [34,35,37,43,44,45,46]. The primary mode of action of inert dusts on insects involves the adsorption of the waxy cuticular layer leading to death by desiccation [1,20,22,47]. Mortality occurs when insects lose more than 60% of their water content from their body due to desiccation [20]. In a study conducted by Rigaur et al. [45], it was demonstrated that adult mortality in *T. castaneum* occurs when the insect’s body loses approximately 30 to 36% of its initial water content of 52–53%. A mortality assessment of *T. castaneum* adults carried out following exposure to silica powders 1 and 2 revealed that the insects became brittle, primarily due to desiccation and a subsequent reduction in the water content of their bodies.

The contact efficacy results showed that *T. castaneum* adults exhibited higher susceptibility to silica powder 1 compared to silica powder 2. We observed complete mortality of *T. castaneum* adults exposed for 36 h to 1.5 to 5 g/m^2^ of silica powder 1. Conversely, complete mortality was achieved when adults were exposed for 48 h to 0.75 to 5 g/m^2^ of silica powder 2. Fields et al. [48] evaluated the efficacy of four diatomaceous earth powders, Dryacide, Insecto, Perma-Guard, and Protect-It, against *T. castaneum* adults at 1.0 g/m^2^ exposed for 24 h followed by placement on untreated wheat for 7 days. They found that Dryacide was the most effective formulation leading to 96% mortality, followed by Protect-It which gave 55% mortality of adults, while the Insecto and Perma-Guard were the least effective, achieving mortalities of 7 and 13%, respectively. Our findings suggest that *T. castaneum* is a relatively tolerant species when it comes to inert dusts, requiring a minimum of 36 h exposure for complete adult mortality. This result is consistent with previous studies that have identified *Tribolium* species as the most tolerant stored product insect species to inert dusts [23,27,31,32,33].

The efficacy of inert dusts against stored product insects can vary based on several factors including the amorphous silica content, particle size, oil adsorption capacity, pH values (<8.5), clay quantity, and other impurities [16,18,23]. The manufacturer-provided elemental composition data for the two powders were related with the mortality response of the two silica powders. Regarding the silicon dioxide content, both powders exceeded 94%. Building on a prior hypothesis derived from our studies with *R. dominica* (Manivannan and Subramanyam, unpublished data), it is presumed that the silicon dioxide levels in silica powders 1 and 2 may have influenced the adsorption of epicuticular lipids from the insect’s integument, ultimately causing death by desiccation [1,49,50]. By comparing the particle diameters of the two silica powders, it was noted that silica powder 1 had a significantly smaller particle size (D_10_, D_50_, and D_90_ values of 4.61, 12.71, and 26.40) in comparison to silica powder 2 (D_10_, D_50_, and D_90_ values of 6.58, 15.84, and 31.43) (Manivannan and Subramanyam, unpublished data). Given this smaller particle size, silica powder 1 had a higher likelihood of adhering to the insect’s body, resulting in increased mortality. This finding aligns with previous research emphasizing the importance of smaller particle sizes for the improved insecticidal activity of inert dust against many insect pests [51,52,53,54,55]. The variation in the effectiveness of the two silica dusts may also arise from differences based on the particle accumulation from grain kernels which adhere to the insect’s body, leading to the adsorption of lipids from the insect’s cuticle [56,57]. However, in the present study, we did not study the adherence of these two silica powders on *T. castaneum* cuticle. Inert dusts with a higher oil adsorption capacity also demonstrated better insecticidal activity [58]. Despite the fact that silica powder 2 had an oil adsorption capacity 0.5 times higher than that of silica powder 1 (Manivannan and Subramanyam, unpublished data), we did not observe silica powder 2 surpassing silica powder 1 in terms of the mortality responses of *T. castaneum* adults.

We achieved complete mortality of *T. castaneum* when the adults were exposed to 5.0 g/m^2^ of silica powder 1 for 20 h. However, complete control of *T. castaneum* was achieved when adults were exposed for 24 h to 6.0 g/m^2^ of silica powder 2, indicating the lower efficacy of silica powder 2 compared to silica powder 1. Tadesse and Subramanyam [59], in studies with non-diatomaceous earth inert dusts from Ethiopia, such as filter cake and Triplex powders, indicated that the complete control of *S. zeamais* adults can be achieved at 7.5 g/m^2^ of filter cake and 10 g/m^2^ of Triplex powders after exposure for 24 h. However, *T. castaneum* is the most tolerant beetle species among stored product insects. The concentrations required for the complete mortality of adults of *T. castaneum* reported in the present study are lower than those observed by Tadesse and Subramanyam [59] in their studies with *S. zeamais.* This difference in the mortality response could be attributed to the lower silicon dioxide contents in filter cake (<54%) and Triplex (<70%) as compared to 94 and 97% observed for silica powders 1 and 2 in our study.

The adult progeny production of *T. castaneum* was inversely related to the powder concentration. We observed that the adult progeny production was completely inhibited when adults were treated with 2.0 to 5.0 g/m^2^ of silica powder 1 for 24 h. On the other hand, a complete inhibition of adult progeny production was observed when adults were treated with 3 to 5 g/m^2^ of silica powder 2 for 36 h. Tadesse and Subramanyam [59] reported complete inhibition of adult progeny production of *S. zeamais* after the exposure of adults to 7.5 to 10 g/m^2^ of filter cake and Triplex powders for 24 h. Complete inhibition of the adult progeny production of *T. castaneum* was observed when adults were exposed to 5.0 g/m^2^ of silica powder 1 and 6.0 g/m^2^ of silica powder 2 for 18 to 24 h. These results further affirmed that silica powder 1, with a smaller particle size diameter, was significantly effective in reducing adult progeny production compared to silica powder 2.

## 5. Conclusions

Our study provides a comprehensive evaluation of the efficacy of silica powders 1 and 2 against *T. castaneum* in concrete arenas simulating empty bin or silo floors. We observed a concentration–time dependent relationship between the application of these inert dusts and *T. castaneum* mortality. Silica powder 1, with its smaller particle size, proved more effective in reducing adult progeny production compared to silica powder 2. Interestingly, despite differences in the oil adsorption capacity, silica powder 1 outperformed silica powder 2 in terms of mortality response and the inhibition of adult progeny production, suggesting that factors beyond oil adsorption capability may play a pivotal role in the insecticidal action of these powders. Our results highlight the potential of these silica powders as a valuable tool for the control of *T. castaneum* for treating empty bin floors prior to the storage of new grains.

## Figures and Tables

**Table 1:** Minimum concentration (g/m^2^) required for 100% mortality of *T. castaneum* exposed to two amorphous silica powders treated concrete arenas for different time periods.

Silica Powder	Concentration (g/m^2^)			
	12 h	24 h	36 h	48 h
Silica 1	_ *	5.0	1.5	1.5
Silica 2	_ *	_ *	_ *	0.75

_ * Complete mortality was not achieved at any of the tested concentrations.

**Table 2:** Pairwise comparison of non-linear models fitted to concentration and 14 d mortality data of *T. castaneum* adults exposed to silica powders 1 and 2.

Powder	Exposure Times Compared	*F*-Value	df	*p*-Value
Silica 1	12 h vs. 24 h	16.4086	2, 20	0.0001 *
	12 h vs. 36 h	23.9973	2, 20	0.0000 *
	12 h vs. 48 h	24.1673	2, 20	0.0000 *
	24 h vs. 36 h	0.9469	2, 20	0.4047
	24 h vs. 48 h	1.2886	2, 20	0.2976
	36 h vs. 48 h	0.0098	2, 20	0.9072
Silica 2	12 h vs. 24 h	7.81	2, 20	0.0031 *
	12 h vs. 36 h	70.49	2, 20	0.0000 *
	12 h vs. 48 h	56.70	2, 20	0.0000 *
	24 h vs. 36 h	55.11	2, 20	0.0000 *
	24 h vs. 48 h	41.54	2, 20	0.0000 *
	36 h vs. 48 h	8.80	2, 20	0.0018 *

* Significant (*p* < 0.05).

**Table 3:** Minimum concentration (g/m^2^) required for complete inhibition of adult progeny production of *T. castaneum* exposed to two amorphous silica powder-treated concrete arenas for different time periods.

Silica Powder	Concentration (g/m^2^)			
	12 h	24 h	36 h	48 h
Silica 1	_ *	2.0	0.75	0.75
Silica 2	_ *	_ *	3.0	0.75

_ * Complete inhibition in adult progeny not achieved at any of the tested concentrations.

**Table 4:** Pairwise comparison of non-linear models fitted to concentration and 42 adult progeny production data of *T. castaneum* adults exposed to silica powders 1 and 2.

Powder	Exposure Times Compared	*F*-Value	df	*p*-Value
Silica 1	12 h vs. 24 h	23.99	2, 20	0.0000 *
	12 h vs. 36 h	12.75	2, 20	0.0003 *
	12 h vs. 48 h	151.74	2, 20	0.0000 *
	24 h vs. 36 h	0.07	2, 20	0.9294
	24 h vs. 48 h	15.52	2, 20	0.0001 *
	36 h vs. 48 h	13.58	2, 20	0.0002 *
Silica 2	12 h vs. 24 h	15.60	2, 20	0.0001 *
	12 h vs. 36 h	127.44	2, 20	0.0000 *
	24 h vs. 36 h	67.44	2, 20	0.0000 *
	24 h vs. 48 h	2173.74	2, 20	0.0000 *
	36 h vs. 48 h	120.13	2, 20	0.0000 *

* Significant (*p* < 0.05).

## Data Availability

Data can be made available upon request.
